# Admiration regulates social hierarchy: Antecedents, dispositions, and effects on intergroup behavior

**DOI:** 10.1016/j.jesp.2012.10.007

**Published:** 2013-05

**Authors:** Joseph Sweetman, Russell Spears, Andrew G. Livingstone, Antony S.R. Manstead

**Affiliations:** Cardiff University, UK

**Keywords:** Collective action, Admiration, Intergroup emotion, Legitimacy, Warmth, Competence

## Abstract

In four studies, we report evidence that admiration affects intergroup behaviors that regulate social hierarchy. We demonstrate that manipulating the legitimacy of status relations affects admiration for the dominant and that this emotion negatively predicts political action tendencies aimed at social change. In addition, we show that greater warmth and competence lead to greater admiration for an outgroup, which in turn positively predicts deferential behavior and intergroup learning. We also demonstrate that, for those with a disposition to feel admiration, increasing admiration for an outgroup decreases willingness to take political action against that outgroup. Finally, we show that when the object of admiration is a subversive “martyr,” admiration positively predicts political action tendencies and behavior aimed at challenging the status quo. These findings provide the first evidence for the important role of admiration in regulating social hierarchy.

## Highlights

► Greater admiration is felt for a group with legitimate status. ► Greater admiration is felt for warm and competent groups. ► Admiration for powerful groups is associated with hierarchy-maintaining behaviors. ► Admiration for “subversive” groups is associated with hierarchy-challenging behaviors. ► Admiration towards an outgroup inhibits hierarchy-maintaining behaviors.

## Introduction

Imagine your personal hero. For many, this simple task may cause a cascade of positive other-praising emotions such as admiration, respect, awe, and reverence. Social psychologists and philosophers have suggested that these emotions play a functional role in maintaining social hierarchy. Specifically, feeling admiration for the powerful and dominant should inhibit challenges to the existing social order. The present research offers the first test of this hypothesis by examining the antecedents and consequences of intergroup admiration, and the role of dispositional admiration-proneness, in predicting hierarchy-enhancing and hierarchy-attenuating intergroup behaviors. In addition to its theorized system-maintaining role, we highlight how admiration can also serve to attenuate intergroup hierarchy, depending on the *object* of one's admiration.

## Admiration regulates social hierarchy

In addition to regulating physiology, perception, and cognition, emotions serve important social functions (Keltner & Haidt, 1999). For example, recent work has begun to sketch the importance of positive emotions in the regulation of interpersonal relationships (Shiota, Campos, Keltner, & Hertenstein, 2004). This regulatory function of emotion also applies at “higher” levels of analyses such as intergroup relations (Iyer & Leach, 2008; Keltner & Haidt, 1999; Mackie, Silver, & Smith, 2004). However, there is little work on the function of positive emotions at this level (although, see Harth, Kessler, & Leach, 2008). Among the positive emotions is a distinct set of other-praising emotions that include admiration, awe, reverence, elevation and respect. These emotions are all associated with appreciating or praising an “other” (Algoe & Haidt, 2009; Keltner & Haidt, 2003; Ortony, Clore, & Collins, 1988). In short, we are likely to feel admiration towards those who engage in praiseworthy actions (Ortony et al., 1988). Philosophers (e.g., Burke, 1757/1990; Smith, 1759/2010) and psychologists (e.g., Henrich & Gil-White, 2001; Keltner & Haidt, 2003) have proposed that admiration plays an important functional role in the maintenance of social hierarchy. More specifically, admiration is thought to carry with it a tendency for the subject of the emotion to defer to the target of the emotion (Burke, 1757/1990; Henrich & Gil-White, 2001; Keltner & Haidt, 2003; Smith, 1759/2010). In this sense, admiration can be seen as maintaining social hierarchy.

A key point of departure between the present approach and previous theorizing on admiration is that we suggest that admiration does not *only* act to maintain the social order. Rather it can also, depending on the *object* of admiration, serve as a means of engendering behavior that challenges the social hierarchy or inhibits behavior that would maintain it. Put simply, we suggest that admiration's role in regulating social hierarchy depends on the object of the emotion (Iyer & Leach, 2008).

## Antecedents, consequences, and dispositions

At the intergroup level, admiration is felt towards groups perceived as being high in competence and warmth (Cuddy, Fiske, & Glick, 2008). Here warmth represents the extent to which groups have shared goals (cooperation), and competence is said to stem from a group's position in the social hierarchy (status). From this stereotype content model's (SCM) perspective, admiration should be associated with helping and cooperating with members of that group (Cuddy et al., 2008). Such a tendency to help the powerful may contribute to the maintenance of the social order. However, given that providing help to a group can in itself serve as a means of creating dependence and reinforcing the recipient's subordinate position (see Nadler, 2002), it seems that intergroup helping is unlikely to be the most important behavior for ensuring that social hierarchy is maintained. Instead, we propose that there are other important behavioral consequences of admiration that help to maintain the social order. Specifically, we propose that admiration maintains social hierarchy by inhibiting political action aimed at challenging the social order, and by engendering deferential behavior.

Our reasoning is in line with approaches that suggest that emotion is crucial to regulating intergroup behavior (e.g., Iyer & Leach, 2008; Mackie et al., 2004). For example, research has shown that anger felt by disadvantaged groups is among the most powerful predictors of political action aimed at changing the social order (Smith & Kessler, 2004; Van Zomeren, Spears, Fischer, & Leach, 2004). We suggest that a focus on action-oriented emotions like anger may obscure an important facet of the regulation of social hierarchy. Rather than social hierarchy being maintained by a *lack* of anger over injustice (Wakslak, Jost, Tyler, & Chen, 2007), we suggest that its maintenance can be more emotionally active. Specifically, admiration for the dominant, as well as a lack of anger, is important for engendering behaviors that facilitate the maintenance of social hierarchy and for inhibiting those that would challenge the social order.

Although intergroup perspectives other than the SCM have not dealt directly with the antecedents and consequences of admiration, many of these other perspectives would suggest that admiration should be elicited by legitimate status and/or power (Jost & Banaji, 1994; Sidanius & Pratto, 1999; Tajfel & Turner, 1979). The legitimate status of the “other” (e.g., outgroup, third-party) should induce admiration. While the idea that legitimacy inhibits political action to challenge the status quo is not new (Tajfel & Turner, 1979), the idea that this is (in part) because of admiration for those that have legitimate status has not previously been directly considered.

Finally, people vary in their tendencies to feel positive emotions, and these dispositions have been found to play an important role in the way people think, perceive, and act (Shiota, Keltner, & Mossman, 2007). Interestingly, dispositions for positive emotions are also related to the regulation of social hierarchy. For instance, research has shown that people who are prone to experience pride are more likely to have been promoted in their jobs or given awards and less likely to have been fired than those who are not prone to experiencing pride (Shiota et al., 2004). We suggest that those that are prone to experience admiration should also, when appraising admiration-eliciting groups, be less likely to take action aimed against that group's progress in the social hierarchy.

## The present research

We aim to demonstrate that admiration plays a crucial role in regulating social hierarchy, and that it does so via its impact on relevant intergroup behaviors. More specifically, we suggest that appraising a group as warm, competent, and having legitimate status should elicit admiration towards that group. Moreover, feeling admiration towards the dominant and powerful will inhibit political action aimed at “progressive” social change. However, when the object of admiration is a subversive hero or martyr, the emotion should engender action to challenge the social order. Similarly, if the object of admiration is an outgroup then admiration may inhibit action aimed at maintaining dominance and engender deferential behavior towards that group. Finally, we suggest that dispositions towards feeling admiration should moderate the relationship between admiration and hierarchy-enhancing intergroup behavior. This reasoning adds to current perspectives on collective political action and social change by placing *positive* emotions at the heart of social hierarchy and suggests that the maintenance (and change) of the status quo relies on positive, as much as negative, emotions. Moreover, it focuses on the way in which emotions inhibit, as opposed to facilitate, different political actions (see also Miller, Cronin, Garcia, & Branscombe, 2009). We tested our ideas by manipulating admiration and its antecedents and examining their influence on political action over income inequality (Study 1), deferential and other intergroup behavior (Study 2), political action against an immigrant group (Study 3), and action for political freedom (Study 4).

## Study 1

In intergroup relations' terms, admiration can be conceptualized as implying an appraisal of legitimate status and/or power. In this study we manipulated the legitimacy of the actions of the object of admiration. More specifically, we manipulated whether “prestigious universities” legitimately (vs. illegitimately) enabled their students to earn more on graduation than those students graduating from “less prestigious universities”.

### Method

#### Participants and design

Participants were 89 undergraduate students (sex and age were not directly recorded, but the modal age was 19 years, and approximately 70% of participants were female) recruited from the participant panel in the School of Psychology at Cardiff University. Participants were randomly assigned to one of two conditions (legitimacy of object's actions: legitimate vs. illegitimate).

#### Procedure

The experiment was presented as a survey of student attitudes towards a research report examining levels of graduate income. Participants were told that the survey was comparing students at different “classes” of university highlighted in the report. We used six outgroup universities, three higher and three lower in status than the participants' institution (although the home institution was not explicitly mentioned in the report). In order to enhance category salience, participants rated students from each class of university on a series of traits relating to the dimensions of warmth and competence. Participants were then instructed to read the report's executive summary. This documented an inequality in graduate earnings between classes of institution, with graduates from prestigious universities earning more on average than their counterparts from less prestigious universities, even after accounting for relevant personal characteristics (e.g., gender, class, age, educational attainment, and employer satisfaction).

In the illegitimate condition, participants read that the inequality was explained by the fact that prestigious universities monopolized the “limited amount of available business and political capital.” As such, through their actions, “prestigious institutions are responsible for the disadvantaged earnings position of graduates from less prestigious universities.” In the legitimate condition, the amount of business and political capital was not described as limited. Rather, inequality was framed as being due to the fact that prestigious universities choose to make businesses/political links, while less prestigious institutions “do not play an active role in establishing and maintaining business and political links,” suggesting that the prestigious universities were not responsible for the plight of disadvantaged students. Participants then went on to complete the rest of the survey incorporating the dependent measures.

#### Measures

##### Manipulation checks

We checked the legitimacy of the prestigious universities' actions using seven items derived from Gordijn, Yzerbyt, Wigboldus, and Dumont (2006). Participants were asked to rate on a scale from 1 (*not at all*) to 7 (*very much*) the extent to which they saw the prestigious universities' actions as: “fair,” “harmless,” “normal,” “rational,” “unjust”(reverse-coded), “prejudicial” (reverse-coded), and “moral” (α = .85).

##### Admiration

We measured admiration towards the prestigious universities by asking participants to what extent they felt: “admiration,” “respect,” “reverence,” “awe,” and “inspiration” when thinking about prestigious universities (α = .92). These items were derived from Algoe and Haidt (2009).

##### Political action tendencies

Participants used a scale from 1 (*very unwilling*) to 7 (*very willing*) to indicate the extent to which they would be willing to perform several actions (α = .92) to support the grievances of graduates from less prestigious universities. The political action items were derived from Van Zomeren et al. (2004): “send an email of protest to the government/MP,” “participate in a demonstration,” “help organize a petition,” “participate in some form of collective action to stop this situation,” “donate money to the cause,” “do something together with others to stop this situation,” and “participate in raising our collective voice to stop this situation.”

### Results and discussion

#### Manipulation checks

An ANOVA with the legitimacy manipulation as a between-participants factor and legitimacy appraisals as the dependent variable revealed a main effect of the manipulation on the perceived legitimacy of the prestigious universities' actions, *F*(1, 89) = 4.51, *p* = .036, η_p_^2^ = .049. Those in the illegitimate condition perceived the prestigious universities' actions as more illegitimate (*M* = 3.89, *SD* = .94) than those in the legitimate condition (*M* = 4.33, *SD* = 1.03).

#### Path analysis

**Fig. 1:**
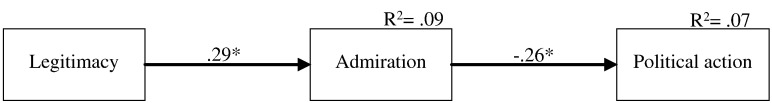
Path-analytic model (Study 1): Influence of legitimacy and admiration on political action tendencies with path weights (*p < .05) and *R*^2^. Model fit: *χ*^2^ = .133, df = 1, *χ*^2^/df = .133, GFI .999, AGFI .994, RMSEA .001.

To test our predictions regarding the indirect effects of our legitimacy manipulation on political action tendencies, we specified the path model illustrated in Fig. 1, which also shows the standardized paths, *R*^2^, and “good” model fit indices (see Schermelleh-Engel, Moosbrugger, & Müller, 2003). The legitimacy manipulation had a significant direct effect on admiration. When prestigious universities' actions were legitimate, participants felt greater levels of admiration for those universities (*M* = 3.96, *SD* = 1.18) than when the actions were illegitimate (*M* = 3.23, *SD* = 1.25). Of importance, there was a significant negative path from admiration to political action. Admiration for the dominant and powerful inhibited political action aimed at social change. Surprisingly, there was no main effect of legitimacy on political action tendencies (β = − .04, *p* = .711). However, we proceeded to test the indirect effects of legitimacy on action tendencies because exogenous variables can exert an influence on the final endogenous variable(s) in a model in the absence of an association (main effect) between them (see Hayes, 2009; Shrout & Bolger, 2002; Zhoa, Lynch, & Chen, 2010).

##### Analysis of indirect effects

To test the indirect effects of the legitimacy manipulation we carried out bootstrapping procedures (see Shrout & Bolger, 2002). This involved generating 5000 random bootstrap samples with replacement from the data set (*N* = 89) and testing the model with these samples. This method does not depend upon a normal sampling distribution (Preacher & Hayes, 2004; Shrout & Bolger, 2002). The analysis revealed a significant indirect effect of legitimacy, with a point estimate of − .075 and a 95% BC (bias-corrected; see Efron, 1987) bootstrap confidence interval of − .169 to − .015. These results are consistent with our predicted causal model. When inequality is explained through legitimate (vs. illegitimate) actions of the dominant party, individuals feel more admiration for the dominant party and are therefore less likely to take action aimed at reducing inequality. This finding supports the notion that admiration is an emotion elicited by legitimate status.

## Study 2

The results of Study 1 are consistent with the idea that legitimate status elicits admiration that helps to maintain the status quo by inhibiting political action aimed at challenging the social order. We wanted to build on these findings in two ways. First, as mentioned above, there are alternative proposals concerning admiration's antecedents and consequences. Cuddy et al. (2008) suggested that admiration is elicited by groups that are seen as high in both warmth and competence, and that this emotion leads to active facilitation (i.e., helping). Therefore, we wanted to examine these two other antecedents. Second, if admiration regulates social hierarchy, it is important to show not only that it inhibits challenges to the social order (i.e., political action) and engenders intergroup helping of the powerful (Cuddy et al., 2008), but also that it engenders *deference* to the object of the admiration (Burke, 1757/1990; Keltner & Haidt, 2003; Smith, 1759/2010). This view is in line with the idea that admiration functions as a means of orientating individuals towards expert models for cultural/social learning, and that this dependency results in those with valued skills obtaining prestige or freely-conferred deference (Henrich & Gil-White, 2001). Third, if our analysis is to add to current perspectives on the regulatory role of emotion in intergroup relations it is important to show that the influence of admiration is unique. Therefore, in the present study we examined the effects of competence and warmth on key SCM emotions (admiration, contempt, and pity) and behavioral tendencies (deference, cultural learning, and helping).

### Method

#### Participants and design

Participants were 123 undergraduate students (17 men and 106 women; age: *M* = 19.44, *SD* = 1.37) recruited from a university participant panel. They were randomly assigned to one of four conditions in a 2 (competence: high vs. low) × 2 (warmth: high vs. low) between-groups factorial design.

#### Materials and procedure

Following Cuddy, Fiske, and Glick (2007), participants read about a fictitious ethnic group expected to immigrate to the UK in the near future. Participants were either informed that, “Members of this group are viewed by their society as *competent* (or *incompetent*) and *intelligent* (or *unintelligent*), and as *warm* (or *not warm*) and *good-natured* (or *not good-natured*).” Participants then went on to complete the rest of the study.

#### Measures

##### Manipulation checks

We checked the perceived warmth and competence of the group by asking participants about the extent to which the immigrant group was thought of as “warm” and “good-natured” (*r* = .88), and “competent” and “intelligent” (*r* = .92).

##### Emotions

Participants were asked how likely they were to feel “contempt,” “pity,” and “admiration” towards the new immigrant group. Participants responded on a 7-point scale ranging from 1 (*extremely unlikely*) to 7 (*extremely likely*).

##### Action tendencies

We asked participants to indicate the extent to which they would behave in each of the following ways towards the new immigrant group: “defer to” (*deference*), “learn from” (*cultural learning*), and “help” (*active facilitation*). Participants responded on a 7-point scale ranging from 1 (*extremely unlikely*) to 7 (*extremely likely*).

### Results and discussion

#### Manipulation checks

We ran an ANOVA with warmth (high vs. low) and competence (high vs. low) as factors and the warmth rating as our dependent measure. Those in the high warmth condition (*M* = 5.49, *SD* = .99) rated the immigrant group as more warm than did those in the low warmth condition (*M* = 2.90, *SD* = 1.35), *F*(1, 120) = 145.08, *p* < .001, η_p_^2^ = .55. There was no main effect of competence, *F*(1, 120) = 0.29, *p* = .59, and the interaction was not significant, *F*(1, 120) = 0.22, *p* = .64. Next we ran a similar ANOVA with competence as our dependent measure. Those in the high competence condition (*M* = 5.41, *SD* = 1.03) rated the immigrant group as more competent than did those in the low competence condition (*M* = 3.00, *SD* = 1.39), *F*(1, 120) = 120.21, *p* < .001, η_p_^2^ = .50. There was no main effect of warmth, *F*(1, 120) = 0.01, *p* = .93, and the interaction was not significant, *F*(1, 120) = 0.47, *p* = .49.

#### Path analysis

##### Full path model

**Fig. 2:**
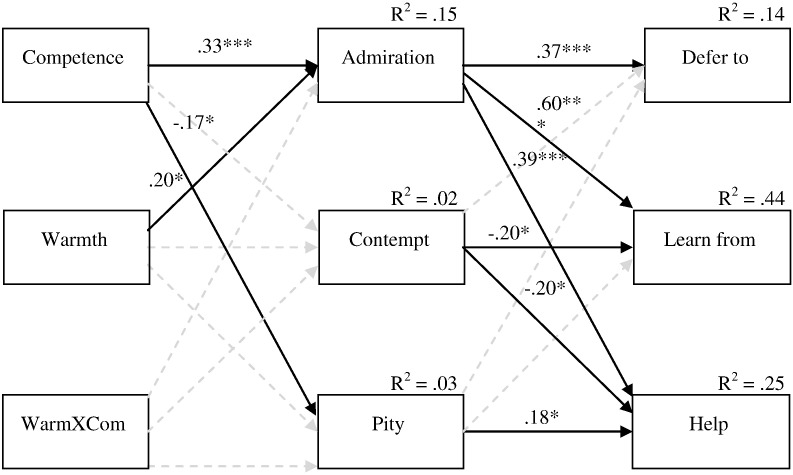
Path-analytic model (Study 2): Influence of competence and warmth on deference, cultural learning, and helping with path weights (*p < .05, **p < .01, ***p < .001) and *R*^2^. Nonsignificant paths are shown as grayed-out broken arrows. Different emotions and action tendencies were allowed to covary with each other. Model fit: *χ*^2^ = 7.864, df = 12, *χ*^2^/df = .655, GFI .986, AGFI .948, RMSEA .001.

In order to test our predictions regarding the effects of competence and warmth we specified the path model shown in Fig. 2. Means, standard deviations, and bivariate correlations between all continuous variables in the model are reported in Table 1. The standardized path weights, *R*^2^, and good model fit indices are shown in Fig. 2. As predicted, there were significant positive direct effects of competence and warmth on admiration, and a significant negative direct effect of competence on pity. When the immigrant group was said to be high in competence, participants felt greater admiration for them. Participants also felt greater admiration when the immigrants were described as high in warmth. We used effect coding to compute the warmth and competence interaction term. This had no significant direct effects on any of the endogenous variables. Greater pity was felt when the immigrant group was described as incompetent, although there was no direct effect of warmth on pity. In addition, there were no direct effects of warmth or competence on contempt. The lack of an effect of warmth on pity and contempt is inconsistent with Cuddy et al.'s correlational data. Given that the authors provided no causal test of the antecedents of intergroup emotions, it may be the case that feelings of pity and contempt affect the perceived warmth of a group.

Consistent with Cuddy et al. (2007) there were significant (positive) direct paths from admiration and pity to helping (active facilitation). In addition, there was a significant negative path from contempt to helping. In terms of our other action tendencies, there was a significant (positive) direct path from admiration to deference. The direct paths from the other emotions to deference were not significant. There was also a significant (positive) path from admiration to cultural learning, and a significant (negative) link between contempt and cultural learning. As predicted, feeling greater admiration (as well as less contempt) for a group was associated with greater likelihood of social learning from that group and (in the case of admiration) with greater levels of deference. This is an initial evidence to support the idea that admiration orientates one towards good models for cultural learning (Henrich & Gil-White, 2001). The idea that learning leads to deference is also supported, in that learning from the immigrant group was positively associated with deference (see Table 1). However, this association did not remain significant in the full model where helping the immigrant group is also included. The findings of this study offer further support for the notion that admiration plays a vital role in regulating social hierarchy, not only by inhibiting challenging behavior (Study 1), but also by encouraging deference to the object of admiration.

#### Analysis of indirect effects

As before, we carried out bootstrapping procedures to test the predicted indirect effects. First, we tested the indirect effects of our manipulated variables on our outcome variables via the pity pathway. This test revealed that the indirect effect of competence on helping was significant, with a point estimate of − .040 and 95% BC CI of − .104 to − .005. When the immigrant group was described as low in competence, participants felt a greater sense of pity, which was associated with greater likelihood of helping the group. There were no other significant indirect effects through pity. In addition, there were no significant indirect effects of our manipulated variables through contempt.

Finally, we tested the indirect effects of our manipulated variables through admiration. This test revealed that the indirect effect of competence on helping was significant, with a point estimate of .139 and 95% BC CI of .072 to .226. There was also an indirect effect of warmth on helping, with a point estimate of .071 and 95% BC CI of .011 to .153. When the immigrant group was described as high in competence or warmth, participants reported greater admiration, which was linked to increased likelihood of helping the group. This is in line with Cuddy et al.'s finding that admiration mediates the positive effects of competence and warmth on helping. Analysis revealed an indirect effect of warmth on deference, with a point estimate of .068 and 95% BC CI of .014 to .142. There was also an indirect effect of competence on deference, with a point estimate of .132 and 95% BC CI of .063 to .231. Here, as the immigrant group's competence or warmth increased, participants' feelings of admiration towards them increased and, in turn, participants were more likely to defer to the group. These results build on the SCM findings (e.g., Cuddy et al., 2007), suggesting that while competence stems from a group's status, it also elicits admiration that, in turn, engenders deference to the group, thereby potentially acting as a means of maintaining status. Admiration not only elicits helping but also simultaneously engenders deference.

Further tests revealed that the indirect effect of competence on cultural learning through admiration was significant, with a point estimate of .215 and 95% BC CI of .122 to .322. There was also an indirect effect of warmth on cultural learning through admiration, with a point estimate of .110 and 95% BC CI of .015 to .222. This supports the idea that the admiration elicited through competence and warmth in turn helps to orientate people towards good models for the cultural learning of skills and competences (Henrich & Gil-White, 2001). Although Henrich and Gil-White (2001) did not explicitly mention warmth as an attribute of their model, given that cooperation is needed for cultural learning it makes sense (all else being equal) that warmer models would be preferred to colder ones.

## Study 3

The results of Studies 1 and 2 shed some light on the antecedents and consequences of intergroup admiration. We wanted to build on these findings in three ways. First, we wanted to provide a more direct test of the causal role of admiration on intergroup behavior. Second, we wanted to examine whether dispositional tendencies to feel admiration influence intergroup behaviors. Third, we wanted to test whether admiration can under some conditions *inhibit* rather than bolster intergroup behaviors that maintain the social hierarchy. Therefore, in the present study we sought to manipulate admiration and to examine the role of individual differences in admiration-proneness on tendencies to take political action against an immigrant group. We reasoned that admiration towards the immigrant group should inhibit political action against it and that this should be particularly true for those that are prone to feeling admiration.

### Method

#### Participants and design

Participants were 58 undergraduate students (3 men and 55 women; age: *M* = 19.55, *SD* = 1.10) recruited from a university participant panel. They were randomly assigned to one of two conditions: admiration vs. control.

#### Materials and procedure

As in Study 2, participants read about a fictitious ethnic group expected to immigrate to the UK in the near future. Following Cuddy et al. (2007), participants were either informed that, “Members of this group are generally admired by others in their society,” or that, “Others in their society generally have no strong feelings towards members of this group.” Participants then went on to complete the rest of the study.

#### Measures

##### Dispositional admiration-proneness

We adapted the dispositional positive emotion scale (Shiota, Keltner, & John, 2006) to form a 12-item (α = .92) measure of admiration-proneness. The items were, “I often feel admiration,” “I see excellence all around me,” “I feel wonder for others achievements almost every day,” “When I see someone performing well, I feel a powerful urge to admire them,” “I have many opportunities to witness skilled and talented people,” “I seek out people that have exceptional ability,” “I admire many people,” “I find it easy to admire others,” “I often notice the amazing things that other people have done,” “The degree of human ability often amazes me,” “I often feel bursts of admiration,” and “On a typical day, others inspire admiration in me.” Participants were asked to rate how true each of the statements were of themselves on a 7-point scale ranging from 1 (*not true*) to 7 (*very true*).

##### Manipulation check

We checked admiration towards the group by asking participants about the extent to which the immigrant group was “admired” and “respected” (*r* = .82).

##### Political action tendencies

Using the same items (α = .96) as Study 1, we asked participants to indicate how willing people would be to engage in political action in order to stop the new immigrant group coming to the UK.

### Results and discussion

We ran an ANOVA with the manipulation check as the DV and the admiration manipulation as the factor and admiration-proneness (mean-centered) entered as a covariate, along with the 2-way interaction term between them. Those in the admiration condition (*M* = 3.54, *SD* = 1.51) did not differ significantly in their ratings of admiration towards the outgroup from the control condition (*M* = 3.08, *SD* = 1.18), *F*(1, 54) = 1.63, *p* = .207. In addition, there was a no main effect of admiration tendencies, *F*(1, 54) = 0.15, *p* = .696. These findings were qualified by a near-significant 2-way interaction, *F*(1, 54) = 3.22, *p* = .078, η_p_^2^ = .06. To interpret the interaction, we performed simple effects analyses of the admiration manipulation at low (− 1 *SD*) and high (+ 1 *SD*) levels of admiration-proneness. Results revealed that when participants were low in admiration-proneness, those in the admiration condition (*M* = 3.28, *SD* = 1.86) did not differ in their admiration reports from those in the control condition (*M* = 3.47, *SD* = 1.92), *F*(1, 54) = 0.13, *p* = .715. In contrast, when participants were high in admiration-proneness, those in the admiration condition (*M* = 3.78, *SD* = 1.81) reported significantly greater levels of admiration towards the immigrant group than those in the control condition (*M* = 2.69, *SD* = 1.94), *F*(1, 54) = 4.76, *p* = .03, η_p_^2^ = .08.

**Fig. 3:**
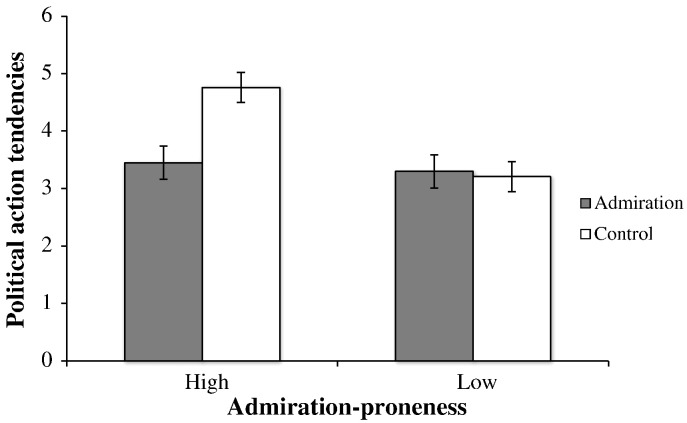
Political action tendencies as a function of admiration and disposition (Study 3). Based on estimated means with high (+ 1SD) and low (− 1SD) admiration-proneness.

Next we ran the ANOVA with political action tendencies as the DV. Results revealed a weak, non-significant, trend with those in the admiration condition (*M* = 3.37, *SD* = 1.52) rating people as less willing to take political action against the immigrant group than did those in the control condition (*M* = 3.91, *SD* = 1.45), *F*(1, 54) = 2.70, *p* = .107, η_p_^2^ = .05. There was a main effect of admiration tendencies, *F*(1, 54) = 5.26, *p* = .026, η_p_^2^ = .09, such that admiration-proneness was positively associated with greater perceived political action tendencies to stop the immigrant group. This finding was unpredicted, but we speculate that in the same way that pride-proneness reflects higher status (Shiota et al., 2004) *general* admiration-proneness may reflect relatively low status and respect for authority. As such, one might expect this emotion disposition to be associated with authoritarian or system-maintaining attitudes. Importantly, these findings were qualified by a near-significant 2-way interaction, *F*(1, 54) = 3.54, *p* = .065, η_p_^2^ = .06. To interpret the interaction, we performed simple effects analyses of the admiration manipulation at low and high levels of admiration-proneness. As can be seen in Fig. 3, when participants were low (− 1 *SD*) in admiration-proneness, those in the admiration condition (*M* = 3.30, *SD* = 1.90) did not differ from those in the control condition (*M* = 3.21, *SD* = 1.82) in terms of political action tendencies against the immigrant group, *F*(1, 54) = 0.03, *p* = .856. In contrast, when participants were high (+ 1 *SD*) in admiration-proneness, those in the admiration condition (*M* = 3.45, *SD* = 1.98) had significantly lower political action tendencies against the immigrant group than those in the control condition (*M* = 4.76, *SD* = 2.16), *F*(1, 54) = 5.78, *p* = .02, η_p_^2^ = .10. As predicted, when presented with a group that was admired (vs. not), those who were prone to feeling admiration rate their group as being less likely to take political action against the outgroup. The notion that admiration-proneness leads to heightened tendencies to distinguish emotion-relevant (i.e., admiration) information about others is in line with the appraisal tendency approach to emotion (Lerner & Keltner, 2000). Although the effect of our manipulation is not straight forward, our findings are consistent and point towards the importance of positive emotion dispositions in the regulation of intergroup relations.

## Study 4

The results of the previous studies yield evidence that admiration towards competent, warm, and high-status groups can inhibit political action aimed at progressive social change, and engender deference. In addition, admiration towards a group can also inhibit political action tendencies aimed against that group. Despite the theoretical emphasis on the social hierarchy maintaining function of admiration, it seems that there is nothing *inherently* hierarchy-maintaining about admiration, at least at the group level. Rather, we argue that it is the object of emotion (see Iyer & Leach, 2008) that determines the social function of admiration. In line with this, we reason that when the object of admiration is a subversive “hero” or “martyr,” admiration should actively engender political action aimed at challenging the social hierarchy. As with much work on collective political action (for a review, see Van Zomeren, Postmes, & Spears, 2008), our previous studies did not employ behavioral measures of political action and employed student samples encountering novel or incidental intergroup situations. What remains to be tested is whether admiration plays a role in historically significant political issues. Therefore, in the present study we aimed to test the impact of admiration towards figures that challenge the status quo (and the authorities) on both political action tendencies and behavior. To do this we examined the emotions of Hong Kong residents in relation to the 1989 Tiananmen Square protests, their willingness to engage in protest, and their decision to sign a petition calling for the Chinese government to change its position on the issue.

### Method

#### Participants and design

Participants in this correlational study were 390 adult Hong Kong residents (154 men and 236 women; age: *M* = 29.03, *SD* = 9.53) who were recruited via advertisements on Facebook. Participants were entered into a prize draw for Amazon vouchers.

#### Procedure

Participants were invited to take part in an online survey of Hong Kong residents' attitudes and feelings regarding the “June 4th incident”^1^ (Tiananmen Square massacre). Participants initially filled in demographic questions and checks to ensure they were Hong Kong residents. They then read details of the suppression of the Tiananmen Square protestors and the resulting substantial loss of life. Participants then read that the Chinese government has not apologized for the killings, refuses to carry out a public inquiry, and interferes with the public mourning of those killed on June 4th. They then went on to complete the key dependent measures, along with several filler items. At the end of the survey participants were informed that they had the chance to participate in an online petition calling on the Chinese government to reverse their position on the June 4th event (i.e., full public investigation and apology, compensation to those affected, and the right of family members to publicly mourn those killed). After this participants were fully debriefed.

#### Measures

##### Anger

Participants were asked how strongly they felt “angry,” “irritated,” and “furious” in relation to “June 4th incident” (α = .84). For all emotion measures, participants responded on a 7-point scale ranging from 1 (*not at all*) to 7 (*extremely*).

##### Sympathy

Participants were asked the extent to which they felt “empathic,” “sympathetic,” and “compassionate” (α = .88) when thinking about those affected by the June 4th incident.

##### Admiration

We measured admiration by asking participants to what extent they felt “admiration,” “respect,” and “inspired” when thinking about the Chinese government (α = .77), and the victims of the June 4th incident (a = .89).

##### Political action

Participants used a scale from 1 (*very unwilling*) to 7 (*very willing*) to indicate their willingness to do each of five things in order to support a campaign to reverse the Chinese government stance on June 4th: “sign a petition addressed to the Chinese government,” “join the annual June 4th protest,” “join the annual June 4th candlelight vigil,” “help organize a petition,” and “donate money to the cause” (α = .95). Protest behavior was measured by recording whether or not participants chose to add their name to the online petition.

### Results and discussion

#### Structural equation modeling (SEM)

**Fig. 4:**
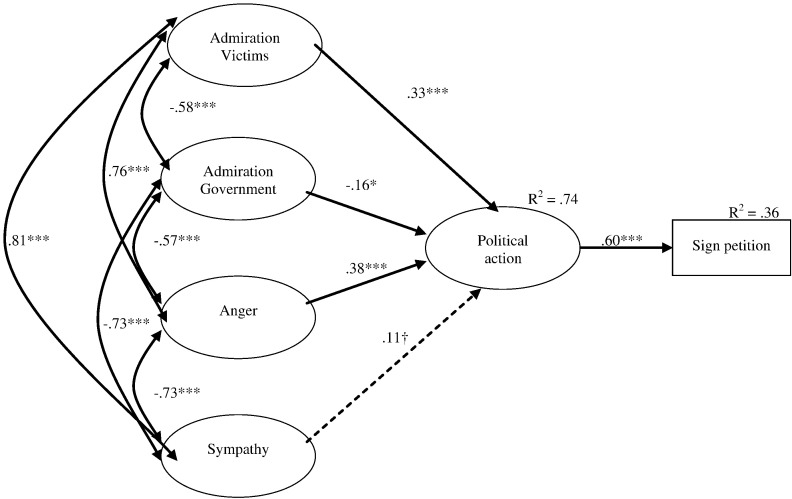
Structural equation model (Study 4): Influence of anger, sympathy, and admiration (towards government and victims) on political action tendencies and signing a petition with path weights (†*p* < .10, *p < .05, **p < .01, ***p < .001) and *R*^2^. Nonsignificant paths are shown as broken arrows. To simplify, manifest variables and the paths from latent to manifest variables are not shown. Model fit: *χ*^2^ = 345.3, df = 125, *χ*^2^/df = 2.762, GFI .911, AGFI .878, RMSEA .067.

The sample size of the present study allowed us to conduct structural equation modeling (using Amos 6.0) using latent variables, which has advantages over path analyses using only manifest variables (Kline, 2005). To test our predictions regarding the effects of admiration (both towards the government and towards the 4th June victims) we specified the model in Fig. 4. Means, standard deviations, and bivariate correlations between all continuous manifest variables in the model are reported in Table 2. The standardized paths, *R*^2^, and adequate model fit statistics are shown in Fig. 4. All manifest measures loaded significantly on to their respective latent variable (ranging between .40 and .96). Analysis revealed that, as expected, there was a significant (positive) direct path from anger to political action tendencies, and a significant (negative) direct path from admiration for the Chinese government to political action tendencies. In addition, there was a significant (positive) direct path from admiration for the victims of June 4th to political action tendencies. Feeling admiration towards the victims of the Tiananmen Square massacre was significantly associated with willingness to take political action aimed at tackling the government's position on the issue, in the presence of anger, sympathy, and admiration for the authorities.

These findings demonstrate the distinct role of admiration in regulating intergroup behavior. Both admiration towards the government and towards the protesters uniquely predicted political action tendencies in the presence of anger and sympathy. In contrast, sympathy was not a significant predictor of political action tendencies. This is consistent with Thomas et al.'s (2009) argument that sympathy is not an optimal emotion for engendering political action. Finally, and as expected, there was a significant (positive) direct path from political action tendencies to signing the petition. The stronger the action tendencies, the more likely participants were to add their name to the petition.

##### Analysis of indirect effects

As before, we carried out bootstrapping procedures to test the predicted indirect effects on our behavioral measure (signing a petition). These revealed that the indirect effect of anger via action tendencies was positive and significant, with a point estimate of .227 and 95% BC CI of .147 to .316. The indirect effect of admiration for the government via action tendencies was negative and significant, with a point estimate of − .099 and 95% BC CI of − .155 to − .046. There was also a positive indirect effect of admiration for the victims of June 4th, via action tendencies, with a point estimate of .196 and 95% BC CI of .111 to .305. The indirect effect of sympathy was not significant.

## General discussion

We set out to examine the antecedents of intergroup admiration, and to test its role in regulating intergroup behaviors that maintain or challenge the social hierarchy. Across four studies, employing a mix of correlational and experimental designs, we found evidence consistent with the idea that admiration regulates social hierarchy. Specifically, legitimate high status engenders admiration that is associated with inhibiting political action aimed at challenging the status quo. In addition, high competence and/or warmth elicit admiration that is associated with deferential behavior. However, we show that admiration does not always have a system-maintaining effect. Rather, admiration towards an outgroup can lead to the inhibition of political action against that group. In addition, admiration can engender political action that challenges the prevailing social order, when the object of admiration is a subversive hero or martyr. Taken together, our findings suggest that positive emotions such as admiration play an important role in regulating social hierarchy through their influence on intergroup behavior (Iyer & Leach, 2008; Mackie et al., 2004).

It is important to distinguish the present approach from other work on affect, emotion, social hierarchy, and political action. For instance, work has shown that maintaining or justifying the system itself may serve a palliative function (Jost & Hunyady, 2002; Wakslak et al., 2007). Here we are not concerned with how justifying the social order may lead to less negative (or more positive) affect. Rather, we are concerned with how specific emotions (DeSteno, Petty, Wegener, & Rucker, 2000; Lerner & Keltner, 2000) involve the tendency to take intergroup actions that regulate the social hierarchy (Mackie et al., 2004). Whereas previous work on collective political action has tended to focus on legitimacy as an antecedent of anger among the disadvantaged (Smith & Kessler, 2004; Van Zomeren et al., 2004), our findings suggest that legitimatization also helps to maintain the social hierarchy not just by decreasing negative emotions like anger (Wakslak et al., 2007), but by engendering positive emotions such as admiration. On the one hand, our results affirm that legitimacy is a core appraisal in the regulation of social hierarchy (Jost & Banaji, 1994; Sidanius & Pratto, 1999; Tajfel & Turner, 1979). On the other hand, our data suggest that the maintenance of social hierarchy may not depend *solely* on legitimacy. Rather, competence may be enough to elicit admiration that, in turn, is associated with deferential behavior. It may be the case that engendering admiration through competence and excellence in valued domains, as opposed to relying on legitimization, is an efficient way for those in a dominant position to maintain their advantage. This use of admiration may represent an indirect or seemingly benign way to maintain social hierarchy (Jackman, 1994). That said, if Henrich and Gil-White (2001) are correct in their contention that admiration leads to freely-conferred dominance, then it may be the case that admiration based on competence serves to legitimize social hierarchy. In other words, legitimacy may be both an antecedent and consequence of admiration. This notion is in line with the idea that emotion leads to a tendency to perceive situations in ways that are consistent with the antecedent cognitive-appraisals of the emotion (Lerner & Keltner, 2000). Future work would do well to examine the nature of this relationship between admiration and legitimacy.

Throughout our studies we chose to focus specifically on admiration because it is the most prototypical other-praising emotion (Ortony et al., 1988). However, we employed a range of other-praising emotion words to measure this emotion. Although this is conceptually and statistically justifiable, we do not believe that all other-praising emotions will be elicited by the same antecedents and have the same kinds of effects on behaviors. While it seems clear that competence and legitimate status engender admiration, it is less clear what engenders the kind of admiration (towards martyrs) that leads to challenges to the social order. After all, the student activists in Tiananmen were not successful in their demands for democracy. Here it seems that warmth and morality (Leach, Ellemers, & Barreto, 2007) may be antecedents of this type of admiration. While emotion theorists have suggested that admiration has a moral component (Haidt, 2003; Haidt & Algoe, 2004), some of the same theorists have more recently suggested that elevation is the other-praising emotion that one feels in response to moral excellence, with admiration reflecting appraisals of competence (Algoe & Haidt, 2009). Future work needs to examine the moral component of other-praising emotions and the way in which they impact on intergroup behaviors that regulate social hierarchy. In addition, future work needs to examine the relationship between admiration for high-status outgroups and tendencies to embrace individual mobility strategies (Tajfel & Turner, 1979) and meritocratic ideologies (Jost, Pelham, Sheldon, & Sullivan, 2003; Sidanius & Pratto, 1999).

Finally, our findings suggest that dispositions to feel admiration may be important in determining the kind of intergroup behaviors that regulate social hierarchy. Indeed, dispositional tendencies to feel positive emotions are differentially linked with peer- and self-rated openness to experience, conscientiousness, extraversion, agreeableness, and neuroticism (Shiota et al., 2006). In the same way, dispositions to feel particular emotions may also be associated with important (intergroup) ideological attitudes such as social dominance orientation and right wing authoritarianism (see Duckitt, Wagner, du Plessis, & Birum, 2002). Future work would do well to examine the relationship between personality, emotional dispositions, and ideological attitudes.

As Adam Smith noted over 250 years ago, we may feel admiration for those in the higher echelons of society, and the effect of this emotion on one's behavior will serve to maintain the social order. However, the present findings suggest that Smith and others were only partly correct in their take on admiration. Admiration for those that choose to challenge the social order may engender insubordination instead of deference. The work reported here goes some way to explaining why and how admiration accomplishes this.

## Figures and Tables

**Table 1:** Bivariate correlations, means and standard deviations for all measures (Study 2).

	1	2	3	4	5
1. Contempt (*M* = 3.12, *SD* = 1.57)					
2. Pity (*M* = 3.41, *SD* = 1.53)	− .17†				
3. Admiration (*M* = 3.22, *SD* = 1.42)	− .14	.13			
4. Defer to (*M* = 2.69, *SD* = 1.29)	.04	− .03	.34⁎⁎⁎		
5. Learn from (*M* = 3.73, *SD* = 1.49)	− .29⁎⁎	− .10	.63⁎⁎⁎	.23⁎⁎	
6. Help (*M* = 4.40, *SD* = 1.48)	− .23⁎	.19⁎	.44⁎⁎⁎	.18⁎	.57⁎⁎⁎

† *p* < .10.

⁎ *p* < .05.

⁎⁎ *p* < .01.

⁎⁎⁎ *p* < .001.

**Table 2:** Bivariate correlations, means and standard deviations for all continuous measures (Study 4).

	1	2	3	4
1. Anger (*M* = 4.83, *SD* = 1.52)				
2. Sympathy (*M* = 4.20, *SD* = .97)	.53⁎⁎⁎			
3. Admiration government (*M* = 2.78, *SD* = 1.50)	− .45⁎⁎⁎	− .23⁎⁎⁎		
4. Admiration victims (*M* = 5.61, *SD* = 1.53)	.68⁎⁎⁎	.58⁎⁎⁎	− .47⁎⁎⁎	
5. Political action (*M* = 4.67, *SD* = 1.84)	.70⁎⁎⁎	.51⁎⁎⁎	− .55⁎⁎⁎	.77⁎⁎⁎

⁎⁎⁎ *p* < .001.

## Footnotes

1 The Tiananmen protest/massacre is referred to as the June 4th “incident” in China.
